# The prevalence of adverse postnatal outcomes for mother and infant in the Netherlands

**DOI:** 10.1371/journal.pone.0202960

**Published:** 2018-09-11

**Authors:** Nynke de Groot, Erwin Birnie, Jolanda H. Vermolen, Jacqueline J. A. Dorscheidt, Gouke J. Bonsel

**Affiliations:** 1 Maternity Care Academic Research Group, Department of Obstetrics and Gynecology, University Medical Center Utrecht, Utrecht University, Utrecht, The Netherlands; 2 Maternity care organization De Waarden, Schoonhoven, The Netherlands; Johns Hopkins School of Public Health, UNITED STATES

## Abstract

**Background:**

In high-income countries delivery usually takes place in a short-stay hospital setting and includes limited specific care after discharge. Perinatal system performance is therefore predominantly expressed in direct terms of delivery outcomes such as preterm birth (PTB), small for gestational age (SGA) or, in case of the mother, perineal rupture and haemorrhage. Additional postnatal complications may emerge, but their incidence is largely unknown. The Dutch obstetric system includes an 8–10 day episode of professional postnatal home maternity care. Our aim was to establish, under routine conditions, the incidence of a comprehensive set of 67 predefined complications and their predictors. A second aim was to address interaction between maternal and child complications.

**Methods:**

The study design was a prospective cohort study of all clients of one large maternity care organization receiving home maternity care in November 2014. We combined maternal background and intrapartum and postnatal characteristics with complication data, routinely recorded by home maternity care assistants. Complication prevalence rates per postnatal day were calculated. Univariate and multivariable logistic regression were used to predict the presence of postnatal complications.

**Results:**

Complications occurred throughout the entire episode of home maternity care and prevalence was high, with 55% of all mother-baby pairs experiencing at least one complication (e.g. cracked nipples, >10% weight loss of the baby) and 5% at least one major complication (e.g. mastitis, cyanosis of the baby). Predictive ability of maternal background and intrapartum and postnatal variables on presence of complications was moderate (max. 62.9%), even when a cumulative risk score was used.

**Conclusions:**

The prevalence rates of maternal and neonatal postnatal complications with care as usual in high-income countries was higher than expected. Professional postnatal follow-up is to be considered in order to timely detect and manage emerging complications with minimal delay. Opportunities for risk-guided care should be investigated further. The pattern of complications in low-income countries remains to be established.

## Introduction

Perinatal care systems are usually evaluated in terms of the direct outcomes of the delivery. Reports on the population level by e.g. the WHO and Peristat use 28-day mortality, rare maternal mortality, haemorrhage, perineal rupture, and, for the baby, congenital anomalies, birth trauma, premature birth (PTB), small for gestational age (SGA), low Apgar score, and hospitalization rates [1–4]. These accounts universally show low adverse outcome rates in high-income countries. While these adverse outcomes are relevant to all countries, the incidence rates in low-income countries are presumably much higher. So far, little attention has been given to the postnatal period. In most middle and high-income countries, women give birth in hospital and are discharged after several days. However, in the first week following delivery, both mother and child can develop complications even if the delivery was uneventful.

Complications that arise in term babies are e.g. feeding problems and/or excess weight loss and icterus, while the mother may suffer from breast problems or infections (among others from CS scar or endometritis). Moreover, adverse delivery outcomes may extend postnatally, like perineal rupture and post-caesarean section complications. Incidence rates of these and other postnatal complications after an uneventful delivery, as well as after a complicated delivery [5,6] or in low resource situations [7] have, however, rarely been reported, and never as a comprehensive report on postnatal complication epidemiology in general. This may hamper even simple guidelines on improving continued breastfeeding, as it is unknown whether low rates depend on complications at the start or complications after initial success.

This paper takes advantage of the Dutch organization of perinatal care which includes use of trained socio-medical professionals (maternity care assistant; MCA) who provide intensive postnatal maternity care at home in the first week after childbirth (98% of all cases). At the initiative of one large maternity care organization (MCO), a comprehensive complication registry was developed and implemented as an extension of the existing quality monitoring system. This study (known as ERKEN-study) is the first report on the 10-day cumulative incidence of 67 predefined maternal and newborn complications, so called ‘critical events’, including 14 major critical events in the postnatal period under routine conditions in a high-income country. The defining feature is not a clinical diagnosis (although in most cases this is present), but the presence of an abnormal condition that requires a professional action from the MCA, in agreement with existing guidelines.

We hypothesized in view of the international practice to discharging women from hospital after a few days that the majority of complications (>90%) would occur within the first 5 days. Additionally, we expected little relation between maternal and neonatal complications. Finally we expected a similar predictability pattern as known for perinatal outcomes in developed countries (parity, low SES).

By describing the epidemiology of the normal postnatal complications by straightforward observational incidence rates, we aimed to set a reference to determine priorities for medical education and care, and to support health policy on postnatal surveillance.

## Methods

### Background

In the Dutch obstetric system, birth takes place either in a short-stay hospital setting or at home (about 20% of all cases). In case of an uneventful hospital birth, discharge usually takes place within 24 hours after birth and ‘home maternity care’ starts thereof. In case of an eventful hospital delivery, discharge and onset of home maternity care is delayed. If the birth already takes place at home, the MCA provides home maternity care immediately after birth. Standard home maternity care, as defined by the health insurance system, implies that the MCA is present for 45–50 hours over a 7–8 day period. The MCA is responsible for the primary clinical (e.g. wound) and psychological care of mother and baby. The MCA also gives instruction to the mother on prevention and self-care, e.g. on breastfeeding, the best approach to persistent crying, and the interpretation of common ailments, thereby raising her empowerment. The MCA also monitors during the day all emerging medical and non-medical critical events. The MCA either handles these critical events independently—guidelines are present—, or refers to other health professionals, especially midwife, obstetrician, or GP.

### Design and population

Our study design was a prospective cohort study from a single maternity care organization (MCO De Waarden [KDW]). KDW is one of the largest providers of home maternity care services in The Netherlands, and covers about 7.5% of all (about 170–180,000) annual births. The catchment area of KDW includes areas of differing degrees of urbanization (i.e. rural, small and large cities) all over the country. From an organizational point of view, KDW, like all large MCOs, consists of regional subunits. KDW commissioned the study as report on quality of care. In our cohort we included anonymized data from all KDW mother-baby pairs that received home maternity care in November 2014. National data indicate that less than 5% of women do not receive maternity care, predominantly for medical reasons (e.g. >10 days NICU stay for very premature neonates or serious congenital disease, and serious maternal complications requiring prolonged hospital admission [8]).

### Outcome measure

The primary outcome measure was the daily incidence rate of so-called “critical events” or complications, as observed by the MCA during home maternity care (further reported as: ‘(MCA reported) complications’, according to medical practice). A MCA-reported complication is any of 67 predefined events, assigned to either the mother, the baby or the family/environment occurring postnatally, that requires professional attention by either the MCA or other (medical) professional (e.g. caesarean section wound infections, fever, skin rash of the baby). Major complications are a predefined subset of serious complications that, according to existing guidelines, require immediate/acute medical attention (e.g. postpartum haemorrhage, cyanosis of the baby). Although major complications are rare, their prompt observation and attention is thought essential, as their implications are profound.

### List of complications

A complication registration form was already into place prior to the study and part of the existing quality system. Monitoring of complications is an obligatory part of the maternity care system. Complete uniform recording by all MCAs is required, including for this study active recording if no complication is observed. To warrant uniform registration and prevent errors in recording, the pre-existing list was ordered into subgroups according to who was involved in the event (i.e. mother, baby, or family), and what body system was involved. The list of mother, baby, and family complications is displayed in S1 and S2 Tables. During the study the presence of any complication, the type and date of observation were recorded.

### Determinants of complications

The determinants were sociodemographic and care process characteristics that are routinely recorded for each mother-baby pair. The determinants were grouped according to the time the information is or becomes available, resulting in a subset of ‘Maternal background’ variables (age, socio-economic status [based on zip-code [9]], ethnicity, parity, type of intake) and ‘Intrapartum and postnatal variables (e.g. referral details, caesarean section, small for gestational age [SGA], preterm birth [PTB], start of home maternity care <24 hours after birth, type of feeding at the start of home maternity care).

### Study process and data collection

During the implementation of ERKEN, maternity care assistants received instructions on the registration process. The key difference with past practice was that 1) a precoded data registry form was used instead of standard note taking in open text fields, 2) date/time details were items that had to be recorded, 3) if no complications were observed, this had to be explicitly recorded, and 4) predefined major complications should all be confirmed using the following validation procedure. The MCA’s supervisor had to verify ex post whether other clinical professionals were promptly involved, and whether measures were put into place that are associated with the event. The process of confirmation required the MCA 1) to call the back office after having addressed the complication, 2) to let the back office verify whether the complication qualified, and 3) to supply, if needed, additional information on symptoms and follow-up actions. Complications were only recorded as major after ex post confirmation. In case of doubt, a complication was not labelled ‘major’. Past practice already included debriefing of the MCA as part of professional responsibility.

Each client had her own complication registry form with a random ID as single identification, allowing anonymous processing for e.g. quality processes and research. Forms were, and still are, digitalized and stored with the client records.

The records used in this report were routinely collected after a two week run-in period of the improved registration had ended. For one month (November 2014) the recording of complications in all mother-baby pairs used the data registry form while a full digital data recording using handheld tablets was prepared. The digitalized anonymous forms were made available to the researchers, together with elementary sociodemographic and care process data, keeping data anonymous. The socio-economic status indicator was based on the client’s ZIP-code, derived from a nationwide reference table of socio-economic status scores by ZIP-code. It is accepted as a powerful and useful proxy in Dutch birth care [10]. The researchers had no access to the clinical data system of KDW or any other personal information of the client. At no stage of the study, the researchers interacted with the commissioner on the data used (e.g. to improve assumed errors or substitute empty fields).

### Analysis

The primary unit of analysis was the mother-baby pair during the episode of home maternity care (in short: the postnatal period). Before the analyses, we excluded the records of twin deliveries (n = 17) to avoid numerator/denominator complexity. We also excluded the records of very premature babies (gestation age <31 weeks [n = 3]), as maternity care usually starts weeks or even months after the delivery. We distinguished between five patterns of postnatal complications: ‘No complications [OO]’, ‘Complication(s) for mother only [MO]’, ‘Complication(s) for baby only [BO]’, ‘Complication(s) for mother and baby [MB]’ and ‘Complication(s) for mother and/or baby [ANY]’. The OO, MO, BO and MB patterns are mutually exclusive; the ANY pattern is the overarching pattern combining the mutually exclusive MO, BO and MB patterns. Because major complications were rare, we did not distinguish between major and non-major complications. For similar reasons, the few family/setting-related complications were included in the maternal complications.

First, socio-demographic- and care characteristics for each of the five patterns were summarized, using conventional descriptive statistics: means and standard deviations (SDs) for continuous variables with normal distributions, medians and IQRs for continuous variables with skewed distributions, and n (%) for nominal/ordinal variables.

Second, the daily incidence rate of postnatal complications was calculated as the number of mother-baby pairs with at least one complication on a particular day (numerator) x 100% divided by the total number of mother-baby pairs receiving home maternity care on that same day (denominator). In case of multiple complications of the same type (mother, baby, family) on the same day, the first complication recorded was used in analysis.

Note that two different time axes exist. The first is the time axis of home maternity care with day 1 as the arrival of mother and baby at home and the start of maternity care. The second is the time axis of postnatal life, with day 1 as the day of birth. Both axes may differ due to postnatal hospitalization or due to delayed onset of maternity care (rare). We used the second option, yielding incidence rates according to number of days after birth. The observed home maternity care period for the analysis sometimes was left-censored (i.e. not all mother-baby pairs start home maternity care direct after birth), and sometimes right-censored if women opted for a shorter period of home maternity care (e.g. minimum care; 24 hours in 6 days instead of 45–50 hours in 8 days).

Third, crude and adjusted associations between presence of complications (dependent variable) and maternal and intrapartum and postnatal characteristics (independent variables) were studied with univariate and multiple binary logistic regression analysis. Results were expressed as crude or adjusted odds ratios (ORs) with 95% confidence intervals.

We used the above-mentioned patterns OO, MO, BO and MB to create contrasts to show which factors were associated with maternal (MO vs. OO; MB vs. OB), neonatal (OB vs. OO; MB vs. MO), maternal-neonatal (MB vs. OO) and overall presence of complications (contrasting ANY vs. OO). For the multiple logistic regressions, predictive ability was expressed as the cumulative proportion of correct predictions that resulted from the sum of expected probabilities compared to the observed number of cases as cut-off.

Finally, we examined the extent to which complications could be predicted by calculating a cumulative risk score for each mother-baby pair within their respective contrasts. This risk score was calculated as follows: 1) The independent variables with an apparent effect on the presence of complications (i.e. adjusted OR <0.80 or >1.25, as obtained in the multivariable logistic regressions) were selected. SGA and PTB were included regardless of OR, as they are known high-risk factors for adverse outcome, and as selection effects are likely to occur within our sample of SGA and PTB babies. 2) For each of the selected variables, a high risk and low risk category was assigned based on the estimated adjusted ORs (e.g. in MO vs. OO: caesarean section: yes, high risk; no, low risk). 3) Finally, we summed the number of ‘high risk’ variables for each mother-baby pair. 4) The resulting risk score was then entered as independent variable in a logistic regression analysis on complications.

Results were interpreted as significant when *p*<0.05 (two-sided). All analyses were performed using SPSS 23.0.

### Medical Ethical Review Board

The analysis was carried out at the request of the MCO for the purpose of quality monitoring and potential improvement of this organization. The description of the postnatal complication epidemiology under routine conditions may help to set a reference to determine priorities for medical education and care, and to support health policy on postnatal surveillance.

The MERB granted exempt from informed consent for the analysis of ERKEN-complications using a fully anonymized dataset from care as usual (MERB protocol nr: 16/119). The co-authors from KDW of this study are not active as caregiver and therefore have no access to the client data.

## Results

In November 2014, 1140 mother-baby pairs received home maternity care by KDW. A total of 20 twin and extremely premature mother-baby pairs (1.75%) were excluded. There were no missing records as the recording of complications is obligatory part of routine care. The study therefore included 1120 mother-baby pairs.

Table 1 shows that the maternal characteristics were about the same for each of the five patterns. Nulliparous mothers were, as expected, slightly underrepresented in the no complication pattern (OO: 36.6%) relative to all other patterns (ANY: 46.3%; MO: 42.3%; BO: 46.7%; MB: 49.3%). A home antenatal intake visit, obligatory in nulliparous women, was also less common in the no complication pattern (OO: 64.4% vs. ANY: 73.4%; MO: 72.7%; BO: 72.3%; MB: 75.2%).

**Table 1:** Socio-demographic and care characteristics of mother-infant pairs according to complication pattern.

Characteristics	Complication pattern				
	Mother or Baby (ANY; n = 636)	Mother only (MO; n = 186)	Baby only (BO; n = 238)	Mother and Baby (MB; n = 212)	None (OO; n = 484)
***Maternal background***					
**Personal**					
Age, mean, y (SD)	30.8 (4.6)	30.4 (4.7)	30.7 (4.6)	31.2 (4.6)	31.2 (4.6)
< 35	78.5	81.7	77.7	76.4	75.2
≥ 35	21.5	18.3	22.3	23.6	24.8
Socioeconomic status (SES), %					
Low (<p20)	11.7	10.8	10.1	14.2	12.0
Middle (p20-p80)	60.0	61.3	61.8	56.9	58.8
High (>p80)	28.3	28.0	28.2	28.9	29.2
Partner present, %	95.9	92.9	97.9	96.2	95.6
Western ethnicity, %	98.1	98.3	98.7	97.1	98,.
Parity, %					
Par 0	46.3	42.3	46.7	49.3	36.6
Par 1–2	49.3	53.1	49.8	45.3	59.8
Par 3+	4.5	4.6	3.6	5.5	3.7
**Care**					
Gestational age at intake, mean, weeks (SD)	34.3 (1.2)	34.3 (1.4)	34.3 (1.2)	34.3 (1.2)	34.4 (1.4)
Intake type, %					
Home visit	73.4	72.7	72.3	75.2	64.4
Phone	26.6	27.3	27.7	24.8	35.6
***Intrapartum and postnatal***					
**Personal, mother**					
Caesarean section, %	14.6	18.3	10.5	16.0	15.7
**Personal, baby**					
Premature birth (32–37 weeks gestation), %	0.8	0.5	1.3	0.5	3.1
Small for Gestational age (<p10), %	10.1	8.6	11.0	10.4	11.8
Hospitalization (postnatal), %	6.1	4.4	7.5	6.1	2.1
Breastfeeding at start home maternity care, %	78.1	75.3	76.1	78.1	69.8
**Care**					
Referral category, %					
Primary care	31.9	24.7	40.3	28.8	29.3
Secondary / Tertiary care	68.1	75.3	59.7	71.2	70.7
Partus assistance by MCA, %	21.7	16.7	28.6	18.4	20.2
Start HMC <24 hours after birth, %	71.8	68.5	78.0	67.5	68.6
Hours of home maternity care received, mean (SD)	47.9 (9.4)	46.8 (9.4)	47.3 (8.4)	49.6 (10.1)	43.8 (8.5)

ANY = Complications for mother or baby; MO = Complications for mother only; BO = Complications for baby only; MB = Complications for mother and baby; OO = No complications; MCA = Maternity care assistant.

The intrapartum and postnatal characteristics were also comparable across all patterns. Mothers who delivered by caesarean section (with implications for maternity care) were overrepresented in the MO-pattern (MO: 18.3% vs. ANY: 14.6%; BO: 10.5%; MB: 16.0%; OO: 15.7%) and the status of being referred from midwife to the gynaecologist in the BO-pattern (BO: 40.3% vs. ANY: 31.9%; MO: 24.7%; MB: 28.8%; OO: 29.3%), both imbalances to be expected. Neonatal adverse outcomes (PTB, SGA) were uncommon, as was re-hospitalization after the start of home maternity care.

Complications were observed in 55.1% of all mother-baby pairs, of which half experienced more than one complication (27.3% of all mother-baby pairs). Major complications were uncommon (4.7% of all mother-baby pairs); multiple major complications extremely rare (0.3% of all mother-baby pairs). Testing for independence of maternal from neonatal complications, showed 32% excess prevalence in the combined MB-pattern (Chi-square: p<0.01); scrutinizing the diagnostic categories did not reveal obvious common underlying pathways (data not shown).

Table 2 shows the incidence rates of types of complications for the total sample and within each pattern. Note that not all complications exist within each pattern. E.g., in the MO-pattern baby related complications are excluded by definition [indicated with–]. Overall, the most prevalent maternal complications were those related to the breasts (13.6%) and abdomen (7.5%). For the baby, the most prevalent complications related to digestion (27.7%) and skin (12.9%). The majority of major complications involved the breasts (mastitis: 26.4%).

**Table 2:** Incidence of maternal, neonatal, and setting complications (Non-major and Major) for the total sample and according to complication pattern.

Complications	Total sample (n = 1120)		Mother or Baby (ANY; n = 636)		Mother only (MO; n = 186)		Baby only (BO; n = 238)		Mother and Baby (MB; n = 212)	
	Non-major	Major	Non-major	Major	Non-major	Major	Non-major	Major	Non-major	Major
**Mother**										
Brain, %	5.4	0.2	9.4	0.3	17.2	0.5	-	-	13.2	0.5
Breast, %	13.6	1.3	23.9	2.2	28.0	3.2	-	-	47.2	3.8
Abdomen, %	7.5	0.8	13.2	1.4	20.4	2.2	-	-	21.7	2.4
Uterus, %	5.5	0.8	9.7	1.4	14.5	3.2	-	-	16.5	1.4
Pelvis, %	4.3	-	7.5	-	11.3	-	-	-	12.7	-
Leg, %	-	0.4	-	0.8	-	2.7	-	-	-	0.0
Bleeding, %	-	0.4	-	0.6	-	1.1	-	-	-	0.9
General, %	2.7	-	4.7	-	6.5	-	-	-	8.5	-
Setting—Home, %	1.6	-	2.8	-	5.9	-	-	-	3.3	-
Setting—Health, %	1.7	-	3.0	-	5.9	-	-	-	3.8	-
**Baby**										
Digestion, %	27.7	-	48.7	-	-	-	67.6	-	70.3	-
Birth Trauma, %	2.0	0.0	3.5	0.0	-	-	3.8	0.0	6.1	0.0
Skin, %	12.9	0.3	22.6	0.5	-	-	27.7	1.3	36.8	0.0
Suspected domestic violence, %	0.2	0.0	0.3	0.0	-	-	0.4	0.0	0.5	0.0
General, %	5.8	0.9	10.2	1.6	-	-	16.0	2.9	12.7	1.4

ANY = Complications for mother or baby; MO = Complications for mother only; BO = Complications for baby only; MB = Complications for mother and baby; ‘–’ = not applicable.

**Fig 1:**
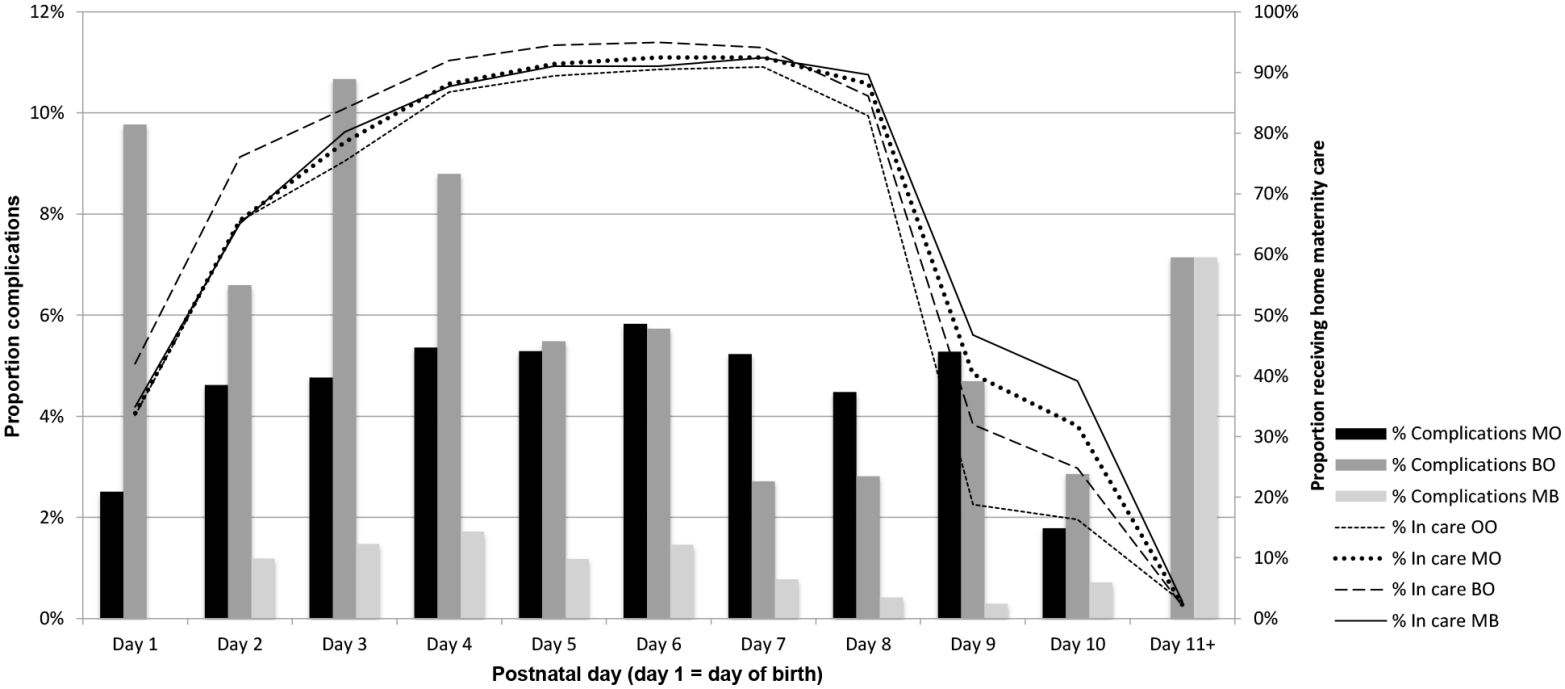
Daily postnatal complications rate (bars) and proportion of mother-baby pairs receiving home maternity care (lines) by postnatal day.

Fig 1 displays the daily postnatal complication rate (bars) and proportion of mother-baby pairs receiving home maternity care (lines) by postnatal day. The primary Y-axis (left) displays the daily proportion of complications by pattern; the secondary Y-axis (right) displays the proportion of mother-baby pairs that received home maternity care at that particular postnatal day for each pattern (100% represents the total group of 1120 mother-baby pairs). For the BO-pattern, the complication rate reaches its peak at 10.7% at day 3 and then steadily declines to 2.9% at day 10. For the MO-pattern, the complication rate increases from 2.5% to 5.8% at day 6, and then steadily declines to 2.8% at day 10. The MB-pattern is comparable to the MO-pattern, except that the overall and daily complication rates are lower.

Overall, complications occur frequently throughout the entire episode of maternity care, with the highest incidence rates on days 1 to 4.

Table 3 shows the results of the univariate regression analysis for each pattern. Type of intake (home vs. phone) and parity (nulliparous vs. multiparous) consistently showed increased odds of complications (home intake: OR range 1.44–1.68; nulliparity: OR range 1.30–1.54). Other explanatory variables did not show a consistent complication effect across pattern. E.g., low socio-economics status (SES <p20) lowered the odds of a neonatal complication (OB vs. OO: OR = 0.80) but increased the odds of a combined mother and child complication (MB vs. OO: OR = 1.22).

**Table 3:** Univariate logistic regression for the impact of maternal background and intrapartum and postnatal variables on presence of complications by complication pattern.

Characteristics	Any vs. None (ANY [n = 636] vs. OO [n = 484])		Mother only vs None (MO [n = 186] vs. OO [n = 484])		Mother and Baby vs. Baby only (MB [n = 212] vs. OB [n = 238])		Baby only vs. None (OB [n = 238] vs. OO [n = 484])		Mother and Baby vs. Mother only (MB [n = 212] vs. MO [n = 186])		Mother and Baby vs. None (MB [n = 212] vs. OO [n = 484])	
	OR	95% C.I.	OR	95% C.I.	OR	95% C.I.	OR	95% C.I.	OR	95% C.I.	OR	95% C.I.
***Maternal background***												
Age (y)												
< 35	1	-	1	-	1	-	1	-	1	-	1	-
≥ 35	0.83	(0.63–1.10)	0.68	(0.44–1.04)	1.08	(0.69–1.67)	0.87	(0.60–1.26)	1.38	(0.85–2.25)	094	(0.64–1.37)
SES												
<p20	0.95	(0.65–1.39)	0.86	(0.49–1.49)	1.53	(0.85–2.76)	0.80	(0.48–1.34)	1.43	(0.77–2.65)	1.22	(0.75–2.00)
p20-80	1	-	1	-	1	-	1	-	1	-	1	-
>p80	0.95	(0.73–1.25)	0.92	(0.63–1.35)	0.73	(0.73–1.70)	0.92	(0.65–1.31)	1.11	(0.71–1.75)	1.02	(0.71–1.48)
Ethnicity												
Western	1	-	1	-	1	-	1	-	1	-	1	-
Non-Western	1.01	(0.42–2.41)	0.86	(0.23–3.22)	2.32	(0.57–9.40)	0.66	(0.18–2.48)	1.79	(0.44–7.26)	1.54	(0.54–4.39)
Parity												
Par 0	1.54	(1.19–1.98)	1.30	(0.91–1.87)	1.16	(0.79–1.71)	1.53	(1.11–2.13)	1.37	(0.90–2.08)	1.54	(1.19–1.98)
Par 1–2	1	-	1	-	1	-	1	-	1	-	1	-
Par 3+	1.49	(0.80–2.80)	1.41	(0.59–3.37)	1.69	(0.65–4.38)	1.17	(0.49–2.78)	1.41	(0.54–3.65)	1.49	(0.80–2.80)
Intake type												
Home visit	1.53	(1.18–1.97)	1.47	(1.01–2.14)	1.17	(0.76–1.78)	1.44	(1.03–2.03)	1.14	(0.73–1.79)	1.68	(1.17–2.42)
Telephone call	1	-	1	-	1	-	1	-	1	-	1	-
***Intrapartum and postnatal***												
Referral category												
Primary care	1	-	1	-	1	-	1	-	1	-	1	-
Secondary care	0.89	(0.69–1.15)	1.26	(0.86–1.86)	1.67	(1.13–2.48)	0.61	(0.44–0.85)	0.81	(0.52–1.27)	1.03	(0.72–1.47)
Caesarean Section												
Yes	0.92	(0.66–1.28)	1.20	(0.77–1.87)	1.63	(0.94–2.83)	0.63	(0.39–1.02)	0.85	(0.51–1.44)	0.91	(0.66–1.59)
No	1	-	1	-	1	-	1	-	1	-	1	-
Small for Gestational Age												
Yes	0.84	(0.58–1.23)	0.71	(0.39–1.26)	0.94	(0.52–1.71)	0.92	(0.56–1.51)	1.23	(0.63–2.42)	087	(0.52–1.46)
No	1	-	1	-	1	-	1	-	1	-	1	-
Premature birth												
Yes	0.25	(0.09–0.69)	0.17	(0.02–1.29)	0.37	(0.04–3.60)	0.40	(0.11–1.39)	0.88	(0.05–14.12)	0.15	(0.02–1.13)
No	1	-	1	-	1	-	1	-	1	-	1	-
Start home maternity care <24 hours after birth												
Yes	1	-	1	-	1	-	1	-	1	-	1	-
No	0.86	(0.66–1.12)	1.00	(0.69–1.45)	1.71	(1.12–2.62)	0.62	(0.43–0.89)	1.05	(0.68–1.61)	0.86	(0.66–1.12)
Breastfeeding at start home maternity care												
Yes	1	-	1	-	1	-	1	-	1	-	1	-
No	0.65	(0.49–0.85)	0.76	(0.52–1.12)	0.65	(0.41–1.04)	0.73	(0.51–1.04)	0.62	(0.38–1.02)	0.47	(0.32–0.71)

ANY = Complications for mother or baby; MO = Complications for mother only; BO = Complications for baby only; MB = Complications for mother and baby; ‘–‘ = not applicable.

The ORs of factors associated with the presence of a combined complication vs. the two single counterparts (MB vs. OB and MB vs. MO) generally differed from the pattern of ORs contrasting the presence of single complication versus none (MO vs. OO and OB vs. OO). Older women (age ≥35y) had an impact of OR 1.08 (ns) in the MB vs. OB contrast but an OR 0.68 (ns) in the MO vs. OO contrast; and MB vs. MO showed OR 1.38 (ns) vs. OB vs. OO showed OR 0.87 (ns). Although none of these associations was significant, these results suggest a degree of interaction between maternal and neonatal complications.

Table 4 displays the results of the multivariable logistic regression analysis across patterns. Results resemble the univariate results, albeit that some ORs decreased in size due to collinearity. The cumulative predictive power of all explanatory variables within each contrast was still moderate at best, with proportions of correct predictions of 55.8% (MO vs. OO contrast), 57.8% (for the MB vs. OB, OB vs. OO, and MB vs. MO contrasts), 60.5% (MB vs. MO contrast), 62.9% (MB vs. OO contrast) and 57.5% (ANY complication vs. OO contrast).

**Table 4:** Multivariable logistic regression for the impact of maternal background, intrapartum and postnatal variables on presence of complications by complication pattern.

Characteristics	Any vs. None^1^ (ANY [n = 636] vs. OO [n = 484])		Mother only vs. None^2^ (MO [n = 186] vs. OO [n = 484])		Mother and Baby vs. Baby only^3^ (MB [n = 212] vs. OB [n = 238])		Baby only vs. None^4^ (OB [n = 238] vs. OO [n = 484])		Mother and Baby vs. Mother only^5^ (MB [n = 212] vs. MO [n = 186])		Mother and Baby vs. None^6^ (MB [n = 212] vs. OO [n = 484])	
	OR	95% C.I.	OR	95% C.I.	OR	95% C.I.	OR	95% C.I.	OR	95% C.I.	OR	95% C.I.
***Maternal background***												
Age (y)												
< 35	1	-	1	-	1	-	1	-	1	-	1	-
≥ 35	0.94	(0.68–1.29)	0.76	(0.48–1.22)	1.00	(0.60–1.65)	1.01	(0.66–1.53)	1.25	(0.72–2.17)	1.04	(0.67–1.61)
SES												
<p20	0.88	(0.57–1.35)	0.71	(0.37–1.37)	1.35	(0.70–2.60)	0.81	(0.54–1.44)	1.60	(0.78–3.29)	1.04	(0.60–1.82)
p20-80	1	-	1	-	1	-	1	-	1	-	1	-
>p80	0.91	(0.68–1.21)	0.91	(0.60–1.39)	1.09	(0.68–1.75)	0.86	(0.58–1.26)	0.95	(0.57–1.57)	0.87	(0.58–1.32)
Ethnicity												
Western	1	-	1	-	1	-	1	-	1	-	1	-
Non-Western	0.92	(0.37–2.31)	0.88	(0.22–3.43)	3.17	(0.75–13.47)	0.58	(0.15–2.28)	1.52	(0.36–6.48)	1.35	(0.45–4.05)
Parity												
Par 0	1.39	(0.10–1.93)	0.99	(0.62–1.56)	1.04	(0.60–1.79)	1.61	(1.04–2.49)	1,68	(0.96–2.94)	1.68	(1.06–2.67)
Par 1–2	1	-	1	-	1	-	1	-	1	-	1	-
Par 3+	1.24	(0.64–2.38)	1.35	(0.54–3.36)	1.14	(0.40–3.22)	1.05	(0.43–2.59)	1.12	(0.40–3.13)	1.41	(0.59–3.37)
Intake type												
Home visit	1.31	(0.94–1.82)	1.40	(0.88–2.23)	0.84	(0.47–1.50)	1.30	(0.84–2.03)	0.83	(0.46–1.49)	1.19	(0.73–1.93)
Telephone call	1	-	1	-	1	-	1	-	1	-	1	-
***Intrapartum and postnatal***												
Referral category												
Primary care	1	-	1	-	1	-	1	-	1	-	1	-
Secondary care	0.86	(0.64–1.17)	1.17	(0.75–1.85)	1.92	(1.19–3.09)	0.60	(0.41–0.87)	0.94	(0.54–1.61)	1.10	(0.71–1.70)
Caesarean Section												
Yes	1.11	(0.68–1.81)	1.55	(0.77–3.13)	0.83	(0.38–1.84)	1.06	(0.53–2.12)	0.58	(0.26–1.30)	1.00	(0.52–1.91)
No	1	-	1	-	1	-	1	-	1	-	1	-
Small for Gestational Age												
Yes	0.89	(0.58–1.37)	0.70	(0.36–1.38)	0.63	(0.31–1.28)	1.21	(0.70–2.09)	119	(0.53–2.65)	0.84	(0.45–1.56)
No	1	-	1	-	1	-	1	-	1	-	1	-
Premature birth												
Yes	0.23	(0.06–0.87)	NO OR		0.35	(0.03–4.26)	0.50	(0.10–2.46)	NO OR		0.20	(0.02–1.65)
No	1	-	1	-	1	-	1	-	1	-	1	-
Start home maternity care <24 hours after birth												
Yes	1	-	1	-	1	-	1	-	1	-	1	-
No	1.00	(0.67–1.49)	0.90	(0.49–1.63)	1.60	(0.84–3.03)	0.81	(0.46–1.40)	1.52	(0.77–3.01)	1.21	(0.72–2.06)
Breastfeeding at start home maternity care												
Yes	1	-	1	-	1	-	1	-	1	-	1	-
No	0.66	(0.49–0.88)	0.70	(0.46–1.07)	0.64	(0.38–1.07)	0.69	(0.47–1.02)	0.70	(0.41–1.21)	0.50	(0.32–0.77)

^1^ Cut-off value 0.568;

^2^ Cutoff value 0.278;

^3^ Cutoff value 0.471;

^4^ Cutoff value 0.330;

^5^ Cutoff value 0.533;

^6^ Cutoff value 0.305.

ANY = Complications for mother or baby; MO = Complications for mother only; BO = Complications for baby only; MB = Complications for mother and baby; OO = No Complications; ‘–‘ = not applicable.

Due to the possible interaction between maternal and neonatal complications, we also calculated the cumulative risk score for the primary contrasts only (MO vs. OO; OB vs. OO; MB vs. OO). The number of selected explanatory variables included in the risk score varied per contrast (range 5–8 out of 11 [see Table 4; selected variables are the ones in grey]). The cumulative risk score showed significantly increased odds of complications for all contrasts (MO vs. OO: OR 1.22; OB vs. OO: OR 1.43; MB vs. OO: 1.19), but the predictive power remained moderate (MO vs. OO: 61.0% correctly predicted; OB vs. OO: 54.2% correctly predicted; MB vs. OO: 52.0% correctly predicted).

## Discussion

This study is to our knowledge the first to investigate the incidence of postnatal complications, as routinely observed by maternity care assistants during home maternity care in the first 10 days after delivery.

One main finding is that the overall complication rate was unexpectedly high, with 55% of all mother-baby pairs reporting at least one maternal or neonatal complication, mostly related to the mothers’ breasts (13.6%) or the baby’s digestion (27.7%). Major complications were uncommon, yet still a sizeable 4.7% (or 1:22) of all mother-baby pairs were affected. Acute mastitis was the most prevalent major complication (1.3%) but we also observed severe and persistent abdominal pain (0.8%), endometritis (0.8%) and an overall poor condition of the baby (i.e. limp/drowsy/measured low temperature, 0.5%).

Secondly, complications occurred throughout the entire episode of home maternity care, with about 2/3s of all recorded complications in the first four days of life, especially for the baby complications. Co-existence of maternal and neonatal complications was 1/3 more common than expected by chance alone. However we could not find obvious clinical pathways. Finally, the predictive power of subsets of maternal background and intrapartum and postnatal variables for complications in general was low; a cumulative risk score yielded slightly better results, still insufficient for risk-guided care.

### Limitations

Our study has several limitations. First, maternity care at home excludes women and/or babies with prolonged hospital stay due to delivery complications or very premature birth. Also perinatal deaths were excluded. Their number is too small to affect the patterns of postnatal complication rates reported here.

Secondly, our cohort included less than expected clients with low socio-economic status (10% compared to 25% nationally) or non-Western background (3% compared to 10% nationally). As the cohort was complete from a registry point of view, this underrepresentation reflects the true average client profile of women who selected KDW as their MCO of choice. In part, this may be the result of selection bias, as home maternity care requires a co-payment. The net result may be that the included women were on average a little more healthy and our findings conservative. The analysis of determinants showed limited predictive power of SES and ethnicity. Hence, we assume the impact of underrepresentation of low SES and non-western ethnicity to be low.

Finally, it is difficult to compare our results, as similar cohort studies in high-income countries with complete registries of predefined complications during the first week after childbirth have not been published, as far as we know. Available evidence focuses on severe or rare morbidity [11,12], developing countries [13,14] and do not apply a cohort design. We could not find textbooks on obstetrics that provide this type of epidemiological information. We assume that low-income countries show substantially higher complication rates of delivery related sequelae in the mother (haemorrhage, infection and sepsis, trauma) and neonatal morbidity in those prematurely born or born after severe fetal stress, in absence of adequate facilities.

### Hypotheses

About 2/3s of all complications occurred within the first four consecutive days after birth. In view of the current organisation of postnatal care focussing on the first postnatal days only, we were surprised by the time-to-event pattern with 1/3 of the complications in the second part of the postnatal period. Arguably, infectious complications need time to develop, as do psychiatric problems. For other complications this ‘delay’, however, is difficult to understand. Mild postnatal complications may so far have escaped epidemiological attention as the focus universally is on delivery and its direct sequelae. No financial incentives are present to delay registration: the health care system includes 8 days of maternity care as standard option with minimal co-payment.

The expected association between nulliparity and intake type (i.e. home visit) and presence of a complication rests on the known increased risks in nulliparous women; part of these postnatal risks actually are excess risks carried forward from an adverse delivery [15]. Surprisingly little impact was observed from socio-economic indicators and ethnicity, particularly if one takes the nulliparity effect into account. The socio-economic effect is on the joint maternal and neonatal incidence rather than the single incidence of maternal or neonatal complications. Perhaps some socio-economically sensitive complications directly after delivery were missed as they occurred within 12 hours, when part of the women is still hospitalized. But even if true, this does not explain the modest effect size.

Altogether we did not expect the complications to be predicted so poorly, regardless which pattern was chosen (mother, baby, or combined). One possibility is that the risk factor set may have been insufficient for two reasons. First, the set may have been incomplete: clinical data of the delivery with a possible impact on the complication rate (e.g. type of pain relief, vacuum extraction) were unavailable, as were lifestyle-related factors. Moreover, the lack of information on obstetric history may affect the prediction of complications with a high recurrence rate between pregnancies (e.g. mastitis, psychiatric complaints, or thrombosis). Also the risk factor set may have been too a specific. Taking the most prevalent complications as an example: for mastitis the most important risk factor is mastitis in a previous pregnancy and for cracked nipples, one of the risk factors is breast engorgement [16,17]; they were not part of the current routine data collection and should perhaps be added to improve its anticipation in postnatal care. We cannot rule out the possibility that complications as such are difficult to predict even if all relevant information would have been available. Evolutionary protection of the mother and the child may restrict the opportunities for simple risk prediction. The interactions between biological, environmental, and psychosocial variables may be too complex to be reduced to straightforward risk relations as used in regression analysis like we did. Use of a cumulative risk score was considered as an alternative approach [18–20], but did not improve predictive ability.

### Implications

Our results in an unselected normal cohort of delivered women show an unexpected high incidence of postnatal complications ranging from very mild (but still requiring some action) to severe. Maternity care assistants in the Netherlands observe and handle these cases at least in first instance. Against common belief this implies a non-trivial medical responsibility for the MCA. While responsibility formally rests with gynaecologists and community midwives, the maternity care system de facto rests on the performance of individual MCAs.

One of the study aims was to contribute to the professionalization of home maternity care. Our results underline the relevance of adequate (medical) training in detecting and handling of common complications. The search for preventive options seems justified in view of the absolute incidence. The current description of professional competencies of MCAs might be elaborated with adjuvant care competencies related to the common complications [21].

Our results may also incite improvement of the current practice of universal home maternity care. Over the last decade, all stakeholders have asked for the provision of maternity care that is more tailored to each specific client. At this stage, the first measure to be taken is the addition of specific risk factor data of common complications to the antenatal intake. Client profiling to allocate the hours of home maternity care or their distribution more efficiently still depends on progress into this direction, and on preventive interventions to reduce the incidence in general.

Finally, our study set-up lends itself to the implementation of this registry in low/middle-income countries, as it depends on careful observation by trained medical personnel with a limited educational level.

### Conclusion

Our study set a benchmark for postnatal complication rates after normal delivery, with 55% of mother-baby pairs experiencing at least one complication, and 4.5% suffering from a major complication. The risk of joint complications in mother and baby is larger than expected on the basis of the singe risks, which suggests interaction. Regrettably, their predictability with the current risk factor set seems moderate, at this stage limiting opportunities for anticipatory or preventive actions. As maternity care assistants have a large share in practical primary postnatal care, education can be adjusted to our findings. The epidemiological findings justify more research into aetiology and the background of combined—yet apparently unrelated—complications.

## Supporting information

S1 Table List of mother and family complications. (PDF)

S2 Table List of baby complications. (PDF)
