# Supplementary material for: The prevalence of adverse postnatal outcomes for mother and infant in the Netherlands
Source: PLoS One. 2018 Sep 11;13(9):e0202960. doi: 10.1371/journal.pone.0202960 (PMC6133278; doi:10.1371/journal.pone.0202960)
Supplement: S1 Table — (PDF) [file pone.0202960.s001.pdf]

| | Maternal and Setting complications | | | |
|---|---|---|---|---|
| **Category** | **Non-major** | **Observations** | **Major** | **Observations** |
| **Brain** | Suspicion of depression | 5 | Suspicion of postpartum psychosis | 2 |
| | Severe psychological derailment (e.g. disorientated, not herself, no hallucinations and approachable) | 10 | | |
| | Insecurity, disproportionate emotional instability | 51 | | |
| **Breast** | Suspicion of fungal infection | 7 | Mastitis | 14 |
| | Other breast problem | 60 | | |
| | Fissures / pain / discomfort | 97 | | |
| **Abdomen** | Inflammation caesarean section wound | 9 | Severe and persistent abdominal | 9 |
| | Cystitis, bladder infection | 40 | | |
| | Other abdomen problem | 42 | | |
| **Uterus** | Fungal infection | 3 | Suspicion of endometritis | 9 |
| | Inflammation stitches | 27 | Prolaps uterus or rectum | 0 |
| | Severe hemorrhoids (persistent pressure, sleep interrupted) | 17 | | |
| | Other uterus problem | 18 | | |
| **Pelvis** | Pain / problems moving | 48 | | |
| **Leg** | | | Thrombosis / Flebitis | 5 |
| **Bleeding** | | | Postnatal hemorrhage | 4 |
| **General** | Cold sore | 10 | | |
| | Fever without other symptoms | 3 | | |
| | Fever with other symptoms | 17 | | |
| **Setting - Home** | Disease other family member | 8 | | |
| | Suspicion of unsafe environment mother | 1 | | |
| | Suspicion of unsafe environment baby | 7 | | |
| | No appropriate physical place mother and / or baby | 4 | | |
| **Setting - Health** | Unknown non-medical risk (e.g. imminent loss of home) | 4 | | |
| | Unknown medical issue prior to conception | 0 | | |
| | Unknown use of medication | 1 | | |
| | Unknown client being seen by general practitioner / specialist | 1 | | |
| | Incorrect use of prescribed medication | 2 | | |
| | Failing to adhere to instructions (feeding, sleeping) | 6 | | |
| | Home birth and/or not seeking specialist care in case of severe risk or birth at <37 weeks | 1 | | |
| | Severe communication problem / miscommunication with other maternity specialists | 5 | | |
| | Severe communication problem with client | 2 | | |
