# Supplementary material for: The prevalence of adverse postnatal outcomes for mother and infant in the Netherlands
Source: PLoS One. 2018 Sep 11;13(9):e0202960. doi: 10.1371/journal.pone.0202960 (PMC6133278; doi:10.1371/journal.pone.0202960)
Supplement: S2 Table — (PDF) [file pone.0202960.s002.pdf]

Category	Neonatal complications			
	Non-major	Number of observations	Major	Number of observations
**Digestion**	Weight loss (>10% of birth weight; less in case of low birth weight or gestational age <37 weeks)	121		
	Suspicion of malnutrition	16		
	Nausea / vomiting (persistent between feedings)	21		
	Thrush	13		
	Problems swallowing / choking	8		
	Other	54		
	Problems initiating breastfeeding	68		
	Tied tongue	34		
	Consult lactation specialist	65		
**Birth Trauma**	Suspicion of fracture (not seen by other professional)	4	Suspicion of cleft lip and/or palate	0
	Suspicion of pareses (not seen by other professional)	0		
	Suspicion of other birth trauma and/or -defect	9		
	Cephalic hematoma	9		
**Skin**	Relevant rash	12	Cyanosis	3
	Icterus	109		
	Other skin problem and/or -defect	32		
**Suspicion of domestic violence**	Negligence	2	Burns	0
			Bruising or other injury	0
**General**	Fever without other symptoms	4	Limp / drowsy / bad condition	5
	Fever with other symptoms	1	Tight chest	3
	Moaning	6	Respiratory assistance / reanimation	2
	Extrem crying behaviour	5		
	Streptococcus	3		
	Low temperature	48		
